# Usefulness of bedside ultrasound in the detection of body packers in the Emergency Department

**DOI:** 10.1186/2036-7902-4-S1-A20

**Published:** 2012-12-18

**Authors:** Tomas Villen, M Garin, J Castañeda, M Sanchez, R Penedo, M Zamorano, D Ly, L Diaz, F Roldan

**Affiliations:** 1 Emergency Ultrasound Division, Emergency Department, Hospital Universitario Ramon y Cajal, Madrid, Spain; 2 Santander-Laredo Family Medicine Training Unit. Spain

## Background

Ultrasound is not used to take part in the clinical approach of body-packers in the Emergency Department. Its usefulness in the evaluation of these kind of patients has not been sufficiently studied. Sometimes, specially in liquid-filled packs, the identification can be specially challenging.

## Objective

To assess the diagnostic accuracy of bedside ultrasound detecting body packers among a small size of body packers and health volunteers.

## Methods

This was a prospective study, in which 29 people were enrolled. In each patient, a 6 seconds retrospective clip of each 9 abdomen anatomical division was recorded. In order to ensure adequate blinding, the clips were reviewed days after its obtention and the reviewer was blinded to all significative data as medical record number or the sonographer that saved the clip. A possitive case consists on the detection of a pack, as an hyperechoic stripe just below the peritoneum line with accoustic shadow and without peristalsis motion and/or air reverberation artifacts. The gold standard was expulsion of at least 10 packs after the scan, and we asume that healthy volunteers enrolled had no packs in their digestive tracts.

## Results

27 of 29 patients enrolled were included to analysis, due to a failure to save all the clips. 15 patients were body packers brought in to the Emergency Department under arrest from Madrid Barajas International Airport, and 12 were healthy volunteers. After the review, the accuracy obtained was (with a 95% Confidence Interval): Sensitivity 0.8 (0.6-1), Specificity 0.5 (0.22-0.78), PPV 0.67 (0.45-0.88), NPV 0.67 (0.36-0.97), PLR 1.6 (0.86-2.97), NLR 0.4 (0.13-1.28).

## Conclusion

The study shows an acceptable value of sensitivity of ultrasound in the body packers detection. Due to the small sample size and the problems related to blinding (mainly impossibility of make pressure over the bowel loops in order to distinguish them from packs) further studies are neccesary to assess the real accuracy of ultrasound among these kind of patients.

